# Early induction and increased risk of precursor B-cell neoplasms after exposure of infant or young-adult mice to ionizing radiation

**DOI:** 10.1093/jrr/rraa055

**Published:** 2020-08-18

**Authors:** Hirotaka Tachibana, Takamitsu Morioka, Kazuhiro Daino, Yi Shang, Mari Ogawa, Misuzu Fujita, Akira Matsuura, Hiroyuki Nogawa, Yoshiya Shimada, Shizuko Kakinuma

**Affiliations:** Department of Radiation Effects Research, National Institute of Radiological Sciences (NIRS), National Institutes for Quantum and Radiological Science and Technology (QST), Chiba 263-8555, Japan; Department of Biology, Graduate School of Science and Engineering, Chiba University, Chiba 263-8522, Japan; Department of Radiation Effects Research, National Institute of Radiological Sciences (NIRS), National Institutes for Quantum and Radiological Science and Technology (QST), Chiba 263-8555, Japan; Department of Radiation Effects Research, National Institute of Radiological Sciences (NIRS), National Institutes for Quantum and Radiological Science and Technology (QST), Chiba 263-8555, Japan; Department of Radiation Effects Research, National Institute of Radiological Sciences (NIRS), National Institutes for Quantum and Radiological Science and Technology (QST), Chiba 263-8555, Japan; Department of Radiation Effects Research, National Institute of Radiological Sciences (NIRS), National Institutes for Quantum and Radiological Science and Technology (QST), Chiba 263-8555, Japan; Department of Radiation Effects Research, National Institute of Radiological Sciences (NIRS), National Institutes for Quantum and Radiological Science and Technology (QST), Chiba 263-8555, Japan; Department of Biology, Graduate School of Science and Engineering, Chiba University, Chiba 263-8522, Japan; Department of Biology, Graduate School of Science, Chiba University, Chiba 263-8522, Japan; Department of Biology, Graduate School of Science and Engineering, Chiba University, Chiba 263-8522, Japan; Department of Biology, Graduate School of Science, Chiba University, Chiba 263-8522, Japan; Executive Director, QST; Chiba 263-8555, Japan; Department of Radiation Effects Research, National Institute of Radiological Sciences (NIRS), National Institutes for Quantum and Radiological Science and Technology (QST), Chiba 263-8555, Japan

**Keywords:** Cancer risk, B-cell neoplasm, Ionizing radiation, Infancy or young adulthood exposure

## Abstract

Epidemiological studies of atomic-bomb survivors have revealed an increased risk of lymphoid neoplasm (i.e. acute lymphoblastic leukemia) associated with radiation exposure. In particular, children are more susceptible to radiation-induced precursor lymphoid neoplasm than adults. Although ~75% of human lymphoid tumors are B-cell neoplasms, the carcinogenic risk associated with each stage of differentiation of B-cells after radiation exposure is poorly understood. Therefore, we irradiated mice at infancy or in young adulthood to investigate the effect of age at exposure on the risk of developing B-cell neoplasms. Histopathology was used to confirm the presence of lymphoid neoplasms, and the population of B-cell neoplasms was classified into the precursor B-cell (pro-B and pre-B cell) type and mature B-cell type, according to immunophenotype. The data revealed that precursor B-cell neoplasms were induced soon after radiation exposure in infancy or young adulthood, resulting in a greater risk of developing the neoplasms. This was particularly the case for the pro-B cell type after young adult exposure. Our findings suggest that exposure to radiation at young age increases the risk of developing precursor B-cell neoplasms in humans.

## INTRODUCTION

Epidemiological studies of atomic-bomb survivors in Hiroshima and Nagasaki have revealed a radiation-related increase in the risk of developing solid and hematopoietic tumors [1–3]. In particular, among atomic-bomb survivors, there was a striking effect of radiation exposure on the development of lymphoid neoplasm (i.e. acute lymphoblastic leukemia) during a short period after exposure [3].

Given the increased use of radiation for medical purposes, such as diagnostic imaging and cancer treatment [4], there is great concern about the onset of secondary cancer after radiation exposure. Indeed, the onset of secondary cancers that can be attributed to radiotherapy has been demonstrated in various tissues including skin and breast [5, 6]. In particular, children are thought to be more sensitive than adults to radiation-induced cancers because their organs and tissues are developing [7]. It has been reported that a relatively younger age at exposure correlates with increased relative risk for developing leukemias excluding chronic lymphocytic leukemia [8]. In addition, several reports have demonstrated that radiotherapy for primary cancer at young age (i.e. <21 years of age) has the potential to induce secondary acute lymphoblastic leukemia [9, 10].

Lymphoid tumors are mainly classified as B-cell and T-cell neoplasms according to immunophenotype, and they are further subclassified as precursor- and mature-cell neoplasms. Previously, we have reported the induction of T-cell neoplasms in mice after radiation exposure at infancy [11, 12]. On the other hand, in a general population, the majority of lymphoid neoplasms are B-cell neoplasms (75%) and the other neoplasms are T-cell neoplasms, including NK-cell neoplasms (5%) and various other known or unknown types (20%) [13]. In addition, it has been reported that the majority of acute lymphoblastic leukemias following radiotherapy at young age are precursor B-cell neoplasms [10]. However, the data concerning the carcinogenic risk associated with each stage of differentiation of B-cells after radiation exposure are still insufficient, and the data concerning exposure at young age are particularly scarce.

In the present study, we investigated the effects of age at radiation exposure on the risk of developing B-cell neoplasms with the precursor- or mature-cell phenotype in mice irradiated with gamma rays during infancy or young adulthood.

## MATERIALS AND METHODS

### Mice and irradiation

F1 hybrid mice were produced by crossing female C57BL/6NCrlCrlj and male C3H/HeNCrlCrlj mice purchased from Charles River Laboratories (Kanagawa, Japan). We assigned certain mice at 3–4 days after birth to three different groups to ensure a similar average body weight among the groups in order to prevent a potential body-weight bias that could possibly influence the carcinogenic effects of radiation exposure [11, 12]. After weaning at 28 days of age, male and female mice were housed together in aluminum cages (up to five mice per cage), and each cage was changed weekly. Mice were provided with wood-shaving bedding and fed a radiation-sterilized diet (MBR-1: Funabashi Farm Co., Tokyo, Japan) and water *ad libitum* (water changed twice weekly). The facility was maintained at 23 ± 3°C with relative humidity 50 ± 10% and a 12-h light–dark cycle. Mice at 1 week of age (1 W, infancy) or 7 weeks of age (7 W, young adulthood) were exposed to a single whole-body dose of 4 Gy gamma rays at 0.5 Gy/min (i.e. at high dose rate) using a ^137^Cs source (Gammacell-40, Nordion, Ottawa, Canada). Mice were monitored daily, and when they became moribund (abnormal posture, respiratory disorders, anemia, etc.) they were euthanized and autopsied for the analyses below. All experimental procedures were conducted according to the Guidelines for Animal Welfare and Experimentation of the National Institute of Radiological Sciences of Japan (No. 07–1017).

### Histopathology

During autopsy, all organs were rapidly fixed with 10% neutral buffered formalin, embedded in paraffin, sectioned transversely at 3–4-μm thickness, and stained with hematoxylin and eosin (HE). Lymphoid neoplasms, including malignant lymphoma and lymphocytic leukemia, were diagnosed by pathologists using J-SHARE (Japan Storehouse of Animal Radiobiology Experiments; NIRS, QST) [14], in which data from autopsy observations and HE specimens prepared by the above methods were registered. A diagnosis of lymphoid neoplasms (for the determination of the possible cause of death) was assigned to cases that showed disordered proliferation of atypical lymphocytes in bone marrow or lymphoid tissues (thymus, spleen, lymph node and Peyer’s patch), and infiltration of malignant lymphocytes into several lymphoid tissues or organs.

### Immunohistochemistry

To classify lymphoid neoplasms on the basis of immunophenotype, tissue sections were subjected to immunohistochemical staining for lymphoid-cell lineage markers including TdT, PAX5, CD45R, IgM and CD3 (Supplementary Table S1, see online supplementary material) [15]. Sections were subjected to immunostaining for TdT or double immunostaining for CD45R and CD3 using the automated immunostaining apparatus Ventana Benchmark Ultra (Roche Diagnostics, Tokyo, Japan) according to the standard program provided by the manufacturer. Immunostaining for PAX5 and IgM was carried out manually according to standard protocols [16]. All sections were counterstained with hematoxylin.

### Classification of lymphoid neoplasms

**Fig. 1.**
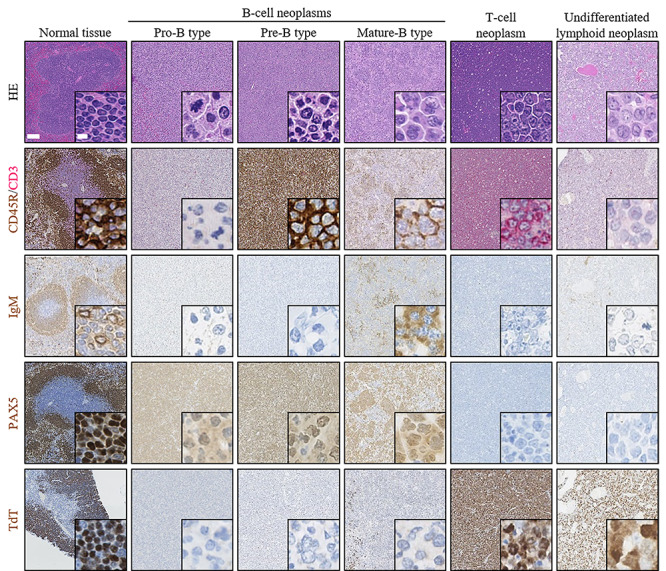
Histopathology and immunohistochemistry of lymphoid neoplasms resected from mice. Each tumor was diagnosed by conventional HE staining. Lymphoid neoplasms were classified as either undifferentiated lymphoid cell or being of B-cell or T-cell origin based on immunohistochemistry. Each B-cell neoplasm was further classified as the pro-B, pre-B or mature-B type. As a positive control, normal spleen was stained for CD45R/CD3, IgM and PAX5, and normal thymus was stained for TdT. Shown are representative images of normal lymphoid tissues (spleen or thymus) and lymphoid neoplasm that had been subjected to HE staining and immunohistochemical staining for CD45R/CD3, IgM, PAX5 and TdT. All images were acquired at the same magnification. Scale bar, 100 μm (5 μm in the inset).

Lymphoid neoplasms were classified into five types on the basis of immunophenotype (Fig. 1) [15]: (i) undifferentiated lymphoid neoplasm (TdT^+^, PAX5^−^, CD3^−^, CD45R^−^), (ii) T-cell neoplasm (CD3^+^, PAX5^−^, CD45R^−^), (iii) pro-B type, poorly differentiated B-cell neoplasm (PAX5^+^, CD3^−^, IgM^−^), (iv) pre-B type, moderately differentiated B-cell neoplasm (CD45R^+^, CD3^−^, IgM^−^) and (v) mature-B type, well-differentiated B-cell neoplasm (IgM^+^, CD3^−^). Lymphoid tumors considered to be mixed (i.e. both CD45R^+^ cells and CD3^+^ cells) or double-positive for PAX5 and CD3 could not be definitively classified as B-cell or T-cell neoplasm and thus were classified as unknown type.

### Statistical analysis

Differences in the incidence of tumors between sex or groups were analyzed with Fisher’s exact test. Mean lifespan differences were analyzed with one-way analysis of variance followed by the *post hoc* Tukey test. Mortality attributable to B-cell or T-cell neoplasms was assessed with Kaplan–Meier survival curves, and any statistically significant differences in survival between groups were determined with the log-rank test. Cox proportional hazard analysis of the risk of developing B-cell neoplasms was performed to calculate hazard ratios for radiation exposure at infancy or in young adulthood. Differences in results were considered significant at *P* < 0.05.

## RESULTS

### Effects of radiation exposure on the incidence of lymphoid neoplasms in mice

**Fig. 2.**
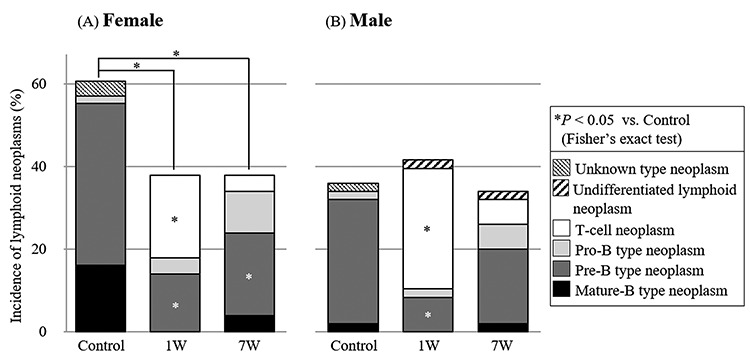
Incidence of each type of lymphoid neoplasm in female (**A**) and male (**B**) mice in the control, 1 W and 7 W groups. Each asterisk denotes a significant difference from the control (*P* < 0.05, pairwise Fisher’s exact test followed by the m × n *x*^2^-square test).

Figure 1 presents representative images of HE and immunohistochemical staining of lymphoid neoplasms. As shown in Fig. 2, the incidence of lymphoid neoplasms was 61% (34 of 56) and 36% (18 of 50) for non-irradiated female and male mice (control), respectively, implying significantly higher incidence of lymphoid neoplasms in female than in male mice. The incidence of lymphoid neoplasms was 38% (19 of 50) for female mice irradiated either during infancy (1 W) or young adulthood (7 W), and it was significantly lower than that of the non-irradiated female mice (Fig. 2A). On the other hand, the incidence of lymphoid neoplasms was 42% (20 of 48) and 34% (17 of 50) in male mice in the 1 W and 7 W groups, respectively, and no significant difference in the incidence of lymphoid neoplasms was observed between the irradiated and non-irradiated groups (Fig. 2B). These results suggest that lymphoid neoplasms spontaneously occur with high frequency in B6C3F1 mice, especially in female mice, and that exposure to radiation does not increase the overall incidence of the neoplasms.

**Table 1:** Experimental design and overall lifespan of mice

Group	Age at irradiation	Dose (Gy)	Sex	Number of mice	Overall lifespan*^a^*
Control	Non-irradiated	0	Female	56	846 ± 139 (345–1210)
			Male	50	829 ± 202 (149–1179)
1 W	1 week	4	Female	50	475 ± 246 (78–864)^*^
			Male	48	505 ± 279 (86–1208)^*^
7 W	7 weeks	4	Female	50	620 ± 206 (198–955)^*^
			Male	50	635 ± 177 (215–941)^*^

^*^a^*^Mean lifespan in days ± SD. Values in parentheses are the minimum and maximum.

^*^Indicates a significant difference vs the control group (*P* < 0.05, one-way analysis of variance followed by *post hoc* pairwise Tukey’s test).

### Effects of radiation exposure and age at exposure on the incidence of each type of lymphoid neoplasm in mice

The incidence of each type of lymphoid neoplasm is also shown in Fig. 2. The female mice in the control group had 2, 39 and 16% (1, 22 and 9 of 56) incidence of the pro-B, pre-B and mature-B type neoplasms, respectively, as well as no incidence of undifferentiated lymphoid or T-cell neoplasms (Fig. 2A). Among female mice in the 1 W group, the incidence of T-cell neoplasm (20%, 10 of 50) was significantly higher than in the control group (0%, 0 of 56). On the other hand, the incidence of pre-B (14%, 7 of 50) and mature-B type (0%, 0 of 50) neoplasms was significantly lower than that in the control group. Likewise, the incidence of pre-B type neoplasm (20%, 10 of 50) among female mice in the 7 W group was significantly lower than that in the control group.

Male mice in the control group had 2, 30 and 2% (1, 15 and 1 of 50) incidence of the pro-B, pre-B and mature-B type neoplasms, respectively, as well as no incidence of undifferentiated lymphoid or T-cell neoplasms (Fig. 2B). Among male mice in the 1 W group, the incidence of T-cell neoplasm (29%, 14 of 48) was significantly higher than in the control group. On the other hand, the incidence of pre-B neoplasm in the 1 W group (8%, 4 of 48) was significantly lower than that in the control group. There were no significant differences in the incidence of each neoplasm in male mice between the 7 W and control groups. These results suggest that radiation exposure during infancy induces T-cell neoplasm in both female and male mice, resulting in decreased incidence of pre-B and mature-B type neoplasms.

### Effects of radiation exposure on the age at death owing to B-cell and T-cell neoplasms in mice

**Fig. 3.**
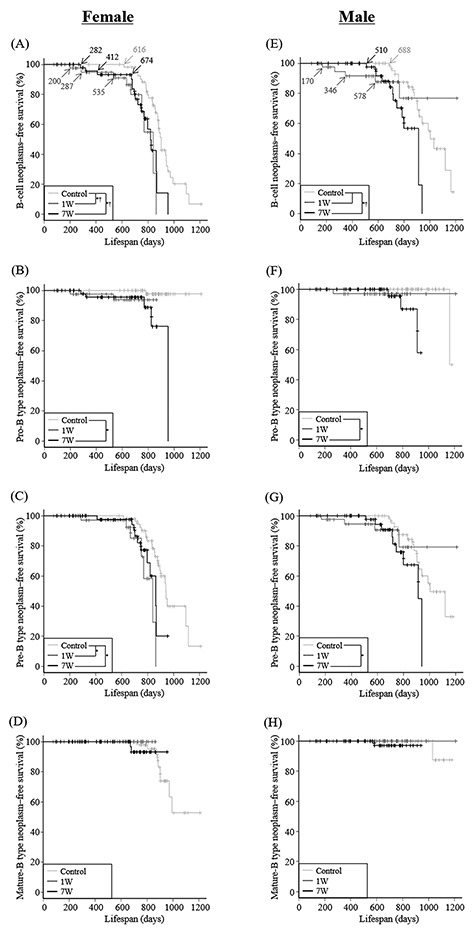
B-cell neoplasms–free survival of female (**A**–**D**) and male (**E**–**H**) mice in the three groups. Kaplan–Meier curves are shown. (A and E) Overall B-cell neoplasms; (B and F) pro-B type; (C and G) pre-B type; and (D and H) mature-B type. Asterisks (in A–H) or daggers (in A and E) denote significant differences between curves during the overall period or late-onset B-cell neoplasm-occurring period.

Table 1 presents data for the overall lifespan of the mice used in this study. Radiation exposure significantly reduced lifespan, regardless of age at exposure. This observation suggested that lymphoid neoplasms, which were the major cause of death in these mice (Fig. 2), developed soon after irradiation. Therefore, we analyzed the lifespan of the mice with lymphoid neoplasms. In female mice, the B-cell neoplasms-free survival rate of the control group began to decrease at 616 days after birth (Fig. 3A). For the 1 W and 7 W groups, the B-cell neoplasms-free survival rate decreased between 200 to 287 days and 282 to 412 days, respectively, after birth (early onset of B-cell neoplasm) and, after a plateau period, it began to decrease again after 535 days and 674 days (late onset of B-cell neoplasm), respectively. The B-cell neoplasms-free survival curves revealed significant differences between the control group and the 1W or 7W group. In addition, the death rate due to late-onset B-cell neoplasms significantly increased in the 1 W and 7 W groups compared with that due to spontaneous B-cell neoplasms in the control group. Likewise, in male mice, the B-cell neoplasms-free survival rate of the control group began to decrease at 688 days after birth (Fig. 3E). For the 1 W group, B-cell neoplasms-free survival rate decreased between 170 and 346 days after birth (early onset of B-cell neoplasm) and, after a plateau period, it began to decrease again after 578 days (late onset of B-cell neoplasm). In contrast, for the 7 W group, early onset of B-cell neoplasm was not observed, and the B-cell neoplasms-free survival rate decreased after 510 days (late onset of B-cell neoplasm). The B-cell neoplasms-free survival curves revealed a significant difference between the 7 W group and the control group. An increase in the death rate due to late-onset B-cell neoplasms in the 7 W group, compared with that due to spontaneous B-cell neoplasms in the control group, was also observed in male mice. Although the mean lifespan of irradiated mice that died from late-onset B-cell neoplasm was not significantly different from control mice that died from spontaneous B-cell neoplasm, the mean lifespan of irradiated mice that died from early-onset B-cell neoplasm was significantly shortened compared with control mice that died from spontaneous B-cell neoplasm (Table 2).

Furthermore, we analyzed the survival curves stratified by the type of B-cell neoplasm. Early-onset pro-B type neoplasm was observed in both the 1 W and 7 W groups (except for the male mice in the 7 W group), and survival curves revealed a significant difference between the 7 W group and the control group (Fig. 3B and F). Early-onset pre-B type neoplasm was also observed in both the 1 W and 7 W groups (except for the male mice in the 7 W group), and survival curves revealed a significant difference in female mice between the 1 W or 7 W and control groups and in male mice between the 7 W and control groups (Fig. 3C and G). In contrast, early-onset mature-B type neoplasm was not observed, and no significant differences in survival curves were observed in both female and male mice among the three groups (Fig. 3D and H). Collectively, these results suggest that radiation exposure accelerates the onset of the pro-B and pre-B type neoplasms. In addition, data for age at death due to pro-B or pre-B-type neoplasms after radiation exposure revealed a bimodal distribution, i.e. mice could be classified as short- or long-lived.

On the other hand, as shown in Supplementary Fig. S1 ( see online supplementary material), T-cell neoplasm-free survival rate decreased between 106 to 348 days and 198 to 226 days in female mice in the 1 W and 7 W groups, respectively, and the survival curves revealed significant differences between the 1 W and control or 7 W group. Likewise, in male mice, T-cell neoplasm-free survival rate decreased between 86 to 682 days and 215 to 387 days in the 1 W and 7 W groups, respectively, and the survival curves revealed significant differences between the 1 W and control or 7 W groups. In addition, T-cell neoplasm was induced significantly earlier than late-onset B-cell neoplasm in the 1 W and 7 W groups, except for the male mice in the 1 W group (Table 2). The induction of T-cell neoplasms in male mice in the 1 W group also showed a trend toward earlier rather than late-onset B-cell neoplasm (*P* = 0.08) (Table 2), due to the fact that several T-cell neoplasm-bearing mice died relatively late (Supplementary Fig. S1). Consistent with our previous reports using infant mice [11, 12], these data suggest that radiation exposure at infancy rapidly and predominantly induces T-cell neoplasms.

**Table 2:** Mean lifespan (days ± SD) of B-cell or T-cell neoplasm-bearing mice

	Female			Male		
	Control	1 W	7 W	Control	1 W	7 W
B-cell neoplasms						
Spontaneous	862 ± 108 (32^a^)	ND	ND	894 ± 140 (17)	ND	ND
	(616–1117^b^)			(688–1163)		
Early-onset	ND^c^	244 ± 62 (2)^*^	337 ± 67 (3)^*^	ND	260 ± 88 (3)^*^	ND
		(200–287)	(282–412)		(170–346)	
Late-onset	ND	724 ± 117 (7)^†^	772 ± 86 (14)^†^	ND	672 ± 133 (2)	733 ± 136 (13) ^†^
		(535–864)	(674–955)		(578–766)	(510–941)
T-cell neoplasm	ND	216 ± 82 (10)	212 ± 20 (2)	ND	249 ± 165 (14)	298 ± 86 (3)
		(106–348)	(198–226)		(86–682)	(215–387)

^*^a^*^The numbers of tumor-bearing mice are shown in parentheses on the upper rows.

^*^b^*^The minimum and maximum lifespans of B-cell or T-cell neoplasm-bearing mice are shown in parentheses on lower rows. ^c^Not diagnosed.

^*^Indicates a significant difference vs B-cell neoplasms in the control group;

^†^indicates a significant difference vs T-cell neoplasm.

**Fig. 4.**
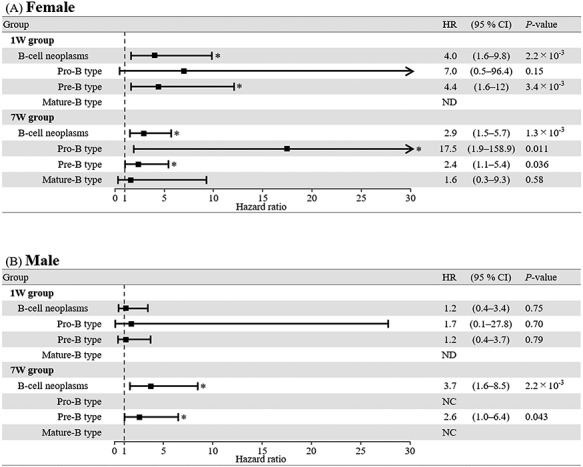
Cox proportional hazard analysis of B-cell neoplasms in female (**A**) and male (**B**) mice. Each asterisk denotes a significant difference from the control (*P* < 0.05). HR = hazard ratio, 95% CI = 95% confidence interval, ND = not diagnosed, NC = not calculated.

### Difference in risk of each type of B-cell neoplasm by age at exposure

We next calculated hazard ratios for lymphoid tumors by radiation exposure. Notably, however, no T-cell neoplasm occurred in the control group (Fig. 2), thus preventing the calculation of hazard ratios for T-cell neoplasms. Figure 4 presents hazard ratios for each type of B-cell neoplasm in the 1 W and 7 W groups vs the control group. In female mice in the 1 W group, the overall hazard ratio for B-cell neoplasms was 4.0 (1.6–9.8) (Fig. 4A). In particular, the hazard ratio for pre-B type neoplasm was 4.4 (1.6–12), implying a significant carcinogenic risk associated with radiation exposure. In addition, although the early-onset pro-B type neoplasm was observed in the 1 W group, no significant carcinogenic risk was observed for pro-B type neoplasm, perhaps due to the low incidence of the neoplasm. Furthermore, in the 7 W group, the overall hazard ratio of B-cell neoplasm was 2.9 (1.5–5.7). In particular, the hazard ratios for the pro-B and pre-B types were 17.5 (1.9–158.9) and 2.4 (1.1–5.4), respectively, implying a significant carcinogenic risk associated with radiation exposure. No significant carcinogenic risk was observed for mature-B type neoplasm.

In contrast, in male mice in the 1 W group, no significant carcinogenic risk was observed for B-cell neoplasms (Fig. 4B). In the 7 W group, the overall hazard ratio for B-cell neoplasms was 3.7 (1.6–8.5). In particular, the hazard ratio for pre-B type neoplasm was 2.6 (1.0–6.4), implying a significant carcinogenic risk associated with radiation exposure. Notably, all male mice in the 7 W group died before the control mice died due to pro-B or mature-B type neoplasm (Fig. 3F and H), thus preventing the calculation of hazard ratios for pro-B or B-cell neoplasm. Collectively, these results suggested that radiation exposure during infancy increases the risk of developing pre-B type neoplasm only in female mice. On the other hand, our data suggest that young adulthood exposure increases the risk of developing B-cell neoplasms (particularly pro-B or pre-B type neoplasms), and this risk is greater in male mice than in female mice.

## DISCUSSION

To reveal the effects of radiation exposure at young age on the development of B-cell lymphoma/leukemia, we investigated the carcinogenic risk of B-cell neoplasms in B6C3F1 mice irradiated with gamma rays at 1 or 7 weeks of age. In mice, the major site of hematopoiesis has already shifted from fetal liver to bone marrow by 1 week of age [17], and this period is analogous to infancy in humans, i.e. ≤1 year of age [18]. On the other hand, in 7-week-old mice, hematopoiesis is carried out in the bone marrow at the same time as sexual maturation and a rapid increase in body weight [11], and this period is analogous to young adulthood in humans, i.e. age range of ~12–21 years [18].

In the present study, we observed a high incidence of B-cell neoplasms in the control group, especially in female mice (Fig. 2). Consistent with our lifespan study, it has been reported that aged B6C3F1 and C57BL/6 (parent of B6C3F1) mice spontaneously develop lymphoid tumors—most of which are B-cell neoplasms—with high frequency, and the incidence of the tumors is higher in female mice than in male mice [19]. Although the genetic backgrounds that lead to the high incidence of spontaneous lymphoid neoplasms in these mice strains are still unknown, the high proportion of B-cell neoplasms seen in these mice is similar to that seen in humans. These mice strains are widely used as a model of human lymphoid neoplasms due to their histopathological and genetic similarities [11, 12, 19–22]. Therefore, the B6C3F1 mice used in this study are a suitable model to investigate differences in the age of onset and the frequency of each immunophenotype (i.e. pro-B, pre-B and mature-B type) of B-cell neoplasms developed spontaneously or after irradiation.

In the present study, death attributable to precursor B-cell (pro-B and pre-B type) neoplasms after radiation exposure during young adulthood (7 W) occurred in mice at a younger age compared with the control group (Figs. 3 and 4). In addition, the risk of developing precursor B-cell neoplasms was increased by radiation exposure during young adulthood, and this was particularly the case for the pro-B type neoplasm. Furthermore, the overall risk of developing B-cell neoplasms was higher in male mice than in female mice. Consistent with our results, an epidemiological study of atomic-bomb survivors revealed that the risk of developing precursor lymphoid neoplasm (i.e. acute lymphoblastic leukemia) was high at younger-attained age [3], although the study does not investigate the immunophenotype of lymphoid neoplasms. In addition, in the same study, the risk of developing precursor lymphoid neoplasm was higher in male survivors compared with female survivors [3]. It has also been reported that the onset of pro-B lymphoid neoplasm is frequently observed in patients following radiotherapy for primary cancers [23]. Thus, these results suggest that radiation exposure during young adulthood accelerates the development of precursor B-cell neoplasms, resulting in a greater risk of developing the neoplasm, despite the decreased incidence of the neoplasms.

Although early-onset precursor B-cell neoplasms were observed in both female and male mice after radiation exposure during infancy (1 W) (Fig. 3B, C, F and G), significant carcinogenic risk was observed only in the pre-B type neoplasm in female mice (Figs. 3C and 4A). Interestingly, consistent with this result, the risk of developing pre-B lymphoid neoplasm is slightly increased in patients who were exposed to diagnostic X-rays at <15 years of age [24]. In accord with our previous reports [11, 12], we also observed that T-cell neoplasm was induced soon after radiation exposure during infancy with high frequency, and the incidence was higher in male than in female mice. Consistent with this, a potential increase in the incidence of T-cell neoplasm has also been reported for patients who have undergone radiation therapy for cancer at <18 years of age [10]. However, the majority of lymphoid neoplasms developed in the patients were precursor B-cell neoplasms [10]. Therefore, present results suggest that T-cell neoplasm is preferentially induced by radiation if a high-dose exposure occurs during infancy (such as in cases of accidental radiation exposure). On the other hand, in mice it has been reported that regeneration of the thymus after high-dose irradiation precedes that of bone marrow and spleen, and subsequently, T-cell neoplasm (i.e. thymic lymphoma) develops from the irradiated thymocytes [25, 26]. Moreover, it has been reported that radiation-induced T-cell neoplasm occurs primarily in young mice rather than old mice, possibly reflecting a difference in the thymic microenvironment at age of exposure [27]. In our study, a large number of mice in the 1 W group, particularly male mice, also died due to the rapid induction of T-cell neoplasm and therefore the proportion of mice that survived until the late period (after 510 days of age) was smaller than in the control group with no induction of T-cell neoplasm (Supplementary Fig. S1). This leads to a lower incidence of late-onset pre-B type neoplasm in the 1 W group compared to the control group (Fig. 3G). This scenario suggests no statistically significant risk for developing pre-B type neoplasm in male mice after radiation exposure during infancy (Fig. 4B). Thus, our results suggest that radiation exposure during infancy potentially increases the carcinogenic risk of precursor B-cell neoplasms, not just for the pre-B type neoplasms in females. On the other hand, it is suggested that a significantly shortened average lifespan of mice in the 1 W group compared to the control group (Table 1) is due to the death of a large proportion of mice from B-cell or T-cell neoplasm in the 1 W group before the death of the majority of mice in the control group (after 600 days of age) (Fig. 3 and Supplementary Fig. S1).

In contrast to the observed risk of precursor B-cell neoplasms, no increase in the risk of mature B-cell neoplasm was observed in mice after radiation exposure (Fig. 4). Indeed, it has been reported that mature B-cell neoplasms, which constitute the majority of non-Hodgkin lymphomas in humans [13], rarely occur in patients after radiotherapy [28]. On the other hand, in the study of atomic-bomb survivors, an excess relative risk of non-Hodgkin lymphoma is reported among young male survivors, although the evidence of a radiation dose response is weak [3]. In the study, however, the types of lymphoma (i.e. precursor B-cell, mature B-cell, T-cell or others) were not investigated. Our results suggest that the observed increased risk of developing non-Hodgkin lymphomas after radiation exposure is due to the early induction of precursor B-cell and T-cell neoplasms in humans. In addition, we hypothesized that radiation-induced precursor B-cell neoplasms are the result of malignant transformation of precursor B-cells or undifferentiated hematopoietic cells (i.e. stem cells) by radiation. Indeed, it has been reported that chromosomal deletion, including a tumor-suppressor gene, was observed in the multipotent progenitor cells in a radiation-induced acute myeloid leukemia mouse model [29].

We found that the age at death due to precursor B-cell neoplasms in irradiated mice showed bimodality at both the early (before 412 days of age) and late (after 510 days of age) periods (Table 2 and Fig. 3). In addition, spontaneous B-cell neoplasms were not observed during the early period, and development of B-cell neoplasms in irradiated mice (i.e. late-onset B-cell neoplasms) was significantly accelerated during the late period compared to development of spontaneous B-cell neoplasms. According to the findings from epidemiological studies, the mechanisms involved in the onset of radiation-induced leukemia are hypothesized to differ with respect to whether the disease is early- or late-onset [2, 30]. That is, early-onset leukemia may be a consequence of transformation of pre-leukemic cells induced by sporadic genomic aberrations that, upon acquisition of additional radiation-induced gene mutations, result in malignant cells [30]. In contrast, late-onset leukemia may develop as a consequence of the acquisition of genomic abnormalities, including gene mutations caused by radiation exposure in normal hematopoietic cells [2]. Therefore, while both early- and late-onset precursor B-cell neoplasms in mice are possibly induced by radiation exposure, their underlying carcinogenic mechanisms may be different.

Our data indicate that radiation exposure induces precursor B-cell lymphoma/leukemia soon after exposure if it occurs at young age, especially during young adulthood, resulting in a greater risk of developing neoplasms. Our results provide valuable information regarding the risk assessment of lymphoid neoplasms following radiation exposure.

## Supplementary Material

Supplemenatry_Figure_S1_rraa055

Supplementary_Table_1_rraa055

## References

[1] Grant EJ, Brenner A, Sugiyama H et al. Solid cancer incidence among the life span study of atomic bomb survivors: 1958-2009. Radiat Res 2017;187:513–37. 28319463 10.1667/RR14492.1 PMC10320812

[2] Richardson D, Sugiyama H, Nishi N et al. Ionizing radiation and leukemia mortality among Japanese atomic bomb survivors, 1950-2000. Radiat Res 2009;172:368–82. 19708786 10.1667/RR1801.1

[3] Hsu WL, Preston DL, Soda M et al. The incidence of leukemia, lymphoma and multiple myeloma among atomic bomb survivors: 1950-2001. Radiat Res 2013;179:361–82. 23398354 10.1667/RR2892.1 PMC3875218

[4] Mettler FA Jr, Thomadsen BR, Bhargavan M et al. Medical radiation exposure in the U.S. in 2006: Preliminary results. Health Phys 2008;95:502–7. 18849682 10.1097/01.HP.0000326333.42287.a2

[5] Meadows AT, Friedman DL, Neglia JP et al. Second neoplasms in survivors of childhood cancer: Findings from the childhood cancer survivor study cohort. J Clin Oncol 2009;27:2356–62. 19255307 10.1200/JCO.2008.21.1920 PMC2738645

[6] Kumar S Second malignant neoplasms following radiotherapy. Int J Environ Res Public Health 2012;9:4744–59. 23249860 10.3390/ijerph9124744 PMC3546788

[7] Brody AS, Frush DP, Huda W et al. Radiation risk to children from computed tomography. Pediatrics 2007;120:677–82. 17766543 10.1542/peds.2007-1910

[8] United Nations Scientific Committee on the Effects of Atomic Radiation Sources, effects and risks of ionizing radiation UNSCEAR. Report United Nations 2013;II:2013.

[9] Friedman DL, Whitton J, Leisenring W et al. Subsequent neoplasms in 5-year survivors of childhood cancer: The childhood cancer survivor study. J Natl Cancer Inst 2010;102:1083–95. 20634481 10.1093/jnci/djq238 PMC2907408

[10] Shivakumar R, Tan W, Wilding GE et al. Biologic features and treatment outcome of secondary acute lymphoblastic leukemia--a review of 101 cases. Ann Oncol 2008;19:1634–8. 18467310 10.1093/annonc/mdn182 PMC2733065

[11] Shang Y, Kakinuma S, Yamauchi K et al. Cancer prevention by adult-onset calorie restriction after infant exposure to ionizing radiation in B6C3F1 male mice. Int J Cancer 2014;135:1038–47. 24482070 10.1002/ijc.28751

[12] Blyth BJ, Kakinuma S, Sunaoshi M et al. Genetic analysis of T cell lymphomas in carbon ion-irradiated mice reveals frequent interstitial chromosome deletions: Implications for second cancer induction in normal tissues during carbon ion radiotherapy. PLoS One 2015;10:e0130666. 26125582 10.1371/journal.pone.0130666 PMC4488329

[13] Morton LM, Wang SS, Devesa SS et al. Lymphoma incidence patterns by WHO subtype in the United States, 1992-2001. Blood 2006;107:265–76. 16150940 10.1182/blood-2005-06-2508 PMC1895348

[14] Morioka T, Blyth BJ, Imaoka T et al. Establishing the Japan-store house of animal radiobiology experiments (J-SHARE), a large-scale necropsy and histopathology archive providing international access to important radiobiology data. Int J Radiat Biol 2019:1–6. 10.1080/09553002.2019.1625458 31145030

[15] Rehg JE, Bush D, Ward JM The utility of immunohistochemistry for the identification of hematopoietic and lymphoid cells in normal tissues and interpretation of proliferative and inflammatory lesions of mice and rats. Toxicol Pathol 2012;40:345–74. 22434870 10.1177/0192623311430695

[16] Morioka T, Miyoshi-Imamura T, Blyth BJ et al. Ionizing radiation, inflammation, and their interactions in colon carcinogenesis in Mlh1-deficient mice. Cancer Sci 2015;106:217–26. 25529563 10.1111/cas.12591 PMC4376429

[17] Mikkola HK, Orkin SH The journey of developing hematopoietic stem cells. Development 2006;133:3733–44. 16968814 10.1242/dev.02568

[18] Cohen Hubal EA, de Wet T, Du Toit L et al. Identifying important life stages for monitoring and assessing risks from exposures to environmental contaminants: Results of a World Health Organization review. Regul Toxicol Pharmacol 2014;69:113–24. 24099754 10.1016/j.yrtph.2013.09.008 PMC5355211

[19] Ward JM Lymphomas and leukemias in mice. Exp Toxicol Pathol 2006;57:377–81. 16713211 10.1016/j.etp.2006.01.007

[20] van der Weyden L, Giotopoulos G, Rust AG et al. Modeling the evolution of ETV6-RUNX1-induced B-cell precursor acute lymphoblastic leukemia in mice. Blood 2011;118:1041–51. 21628403 10.1182/blood-2011-02-338848 PMC3622520

[21] Daino K, Ishikawa A, Suga T et al. Mutational landscape of T-cell lymphoma in mice lacking the DNA mismatch repair gene Mlh1: No synergism with ionizing radiation. Carcinogenesis 2019;40:216–24. 30721949 10.1093/carcin/bgz013

[22] Cheng Y, Chikwava K, Wu C et al. LNK/SH2B3 regulates IL-7 receptor signaling in normal and malignant B-progenitors. J Clin Invest 2016;126:1267–81. 26974155 10.1172/JCI81468 PMC4811117

[23] Ishizawa S, Slovak ML, Popplewell L et al. High frequency of pro-B acute lymphoblastic leukemia in adults with secondary leukemia with 11q23 abnormalities. Leukemia 2003;17:1091–5. 12764373 10.1038/sj.leu.2402918

[24] Shu XO, Potter JD, Linet MS et al. Diagnostic X-rays and ultrasound exposure and risk of childhood acute lymphoblastic leukemia by immunophenotype. Cancer Epidemiol Biomarkers Prev 2002;11:177–85. 11867505

[25] Decleve A, Gerber GB, Leonard A et al. Regneration of thymus, spleen and bone marrow in x-irradiated AKR mice. Radiat Res 1972;51:318–32. 5050463

[26] Sado T, Kamisaku H, Kubo E. Bone marrow-thymus interactions during thymic lymphomagenesis induced by fractionated radiation exposure in B10 mice: Analysis using bone marrow transplantation between thy 1 congenic mice. J Radiat Res 1991; 32 Suppl 2: 168–80. 10.1269/jrr.32.supplement2_168 1823353

[27] Utsuyama M, Hirokawa K Radiation-induced-thymic lymphoma occurs in young, but not in old mice. Exp Mol Pathol 2003;74:319–25. 12782021 10.1016/s0014-4800(03)00026-1

[28] Arslan C, Ozdemir E, Dogan E et al. Secondary hematological malignancies after treatment of non-metastatic breast cancer. J BUON 2011;16:744–50. 22331732

[29] Verbiest T, Finnon R, Brown N et al. Tracking Preleukemic cells in vivo to reveal the sequence of molecular events in radiation Leukemogenesis. Leukemia 2018;32:1435–44. 29556020 10.1038/s41375-018-0085-1 PMC5990525

[30] Nakamura N A hypothesis: Radiation-related leukemia is mainly attributable to the small number of people who carry pre-existing clonally expanded preleukemic cells. Radiat Res 2005;163:258–65. 15733032 10.1667/rr3311

