# Supplementary figures and images for: Early induction and increased risk of precursor B-cell neoplasms after exposure of infant or young-adult mice to ionizing radiation
Source: J Radiat Res. 2020 Aug 18;61(5):648–56. doi: 10.1093/jrr/rraa055 (PMC7482158; doi:10.1093/jrr/rraa055)

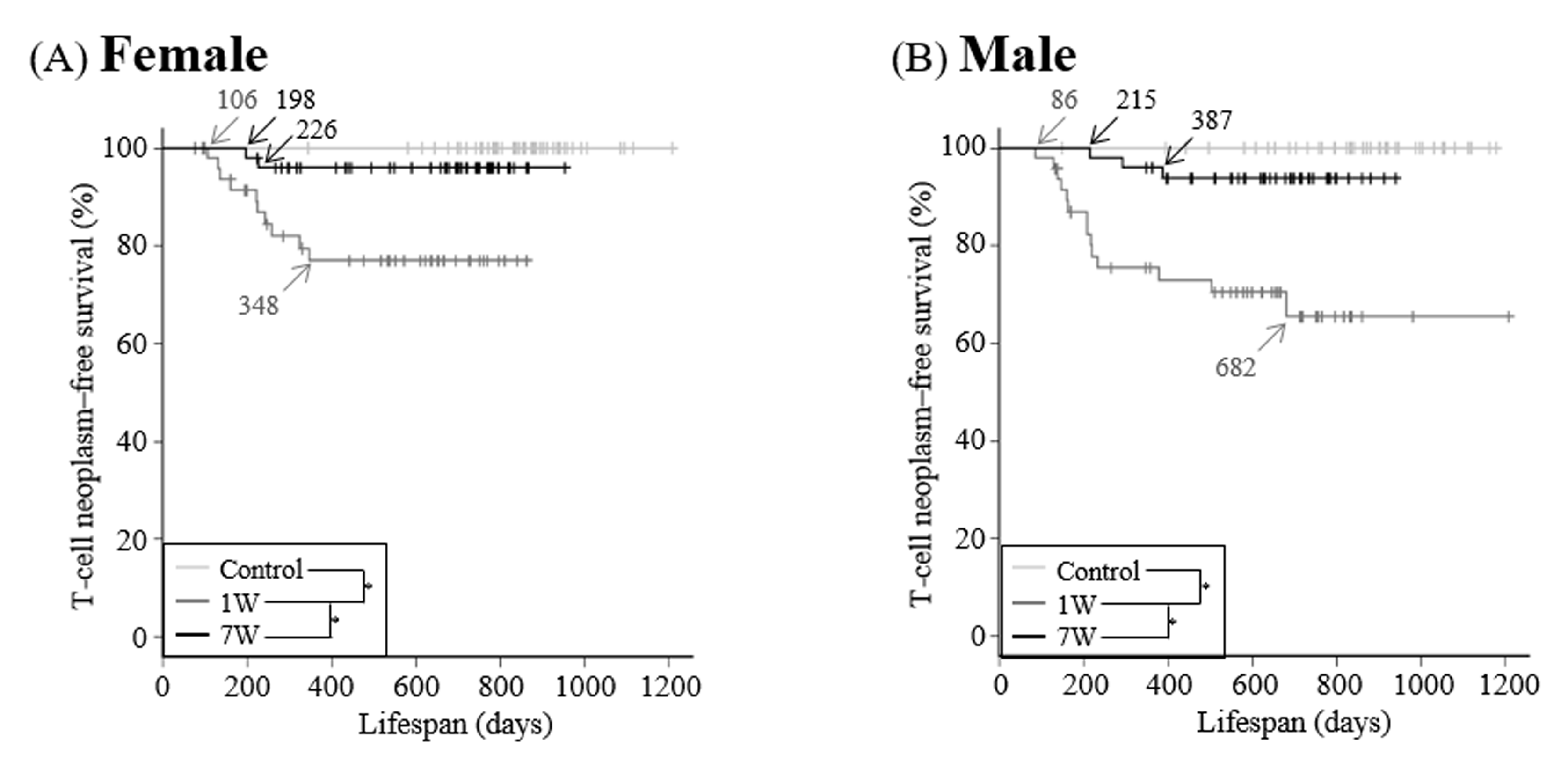

Supplement: Supplemenatry_Figure_S1_rraa055 [file supplemenatry_figure_s1_rraa055.png]
