# Supplementary material for: Early induction and increased risk of precursor B-cell neoplasms after exposure of infant or young-adult mice to ionizing radiation
Source: J Radiat Res. 2020 Aug 18;61(5):648–56. doi: 10.1093/jrr/rraa055 (PMC7482158; doi:10.1093/jrr/rraa055)
Supplement: Supplementary_Table_1_rraa055 [file supplementary_table_1_rraa055.doc]

**Supplementary Table S1.** Summary of immunohistochemical staining methods.

| Marker | Major positive cells | Antibody | Clone (source) | Epitope retrieval | Dilution | Coloring |
| --- | --- | --- | --- | --- | --- | --- |
| TdT*^a^* | Lymphoblasts of B-cell and T-cell lineage | Rabbit-anti human polyclonal | - (Spartech’s) | HIER*^b^* with Tris-EDTA (pH 8.5) | 1:50 | DAB*^c^* |
| Pax5 | Pro-B cell, pre-B cell, and B-cell | Rabbit-anti mouse monoclonal | 3852-1  EPR3730(2) (Abcam) | HIER with Citrate buffer (pH 7.0) | 1:500 | DAB |
| CD45R*^a^*,*^d^* (B220) | Pre-B cell and B-cell | Rat-anti mouse monoclonal | RA3-6B2  (BD Biosciences) | HIER with Tris-EDTA (pH 8.5) | 1:50 | DAB |
| IgM | B-cell | Rat-anti mouse monoclonal | II/41 (Novus Biologicals) | EIER*^e^* with Proteinase-K | 1:200 | DAB |
| CD3*^a^*,*^d^* | T-cell lineage | Rabbit-anti mouse monoclonal | SP7 (Abcam) | HIER with Tris-EDTA (pH 8.5) | 1:150 | Fast red |

*^a^* Semi-automatic staining with the Ventana benchmark ultra.

*^b^* HIER, heat-induced epitope retrieval method.

*^c^* DAB, 3',3'-diaminobenzidine.

*^d^* Double staining.

*^e^* EIER, enzyme-induced epitope retrieval method.
